# Strengthening natural hazard preparedness in Nepal: insights from stakeholder and community engagement

**DOI:** 10.1080/16549716.2026.2692889

**Published:** 2026-06-24

**Authors:** Santosh Bhatta, Julie Mytton, Sunil Kumar Joshi, Puspa Raj Pant, Pratik Adhikary, Basanta Raj Adhikari, Suraj Gautam, John Powell, Edwin van Teijlingen

**Affiliations:** a School of Health and Social Wellbeing, College of Health Science and Society, University of the West of England, Bristol, UK; b Nepal Injury Research Centre, Kathmandu Medical College, Kathmandu, Nepal; c Centre for Disaster Studies, Institute of Engineering, Tribhuvan University, Pulchowk, Nepal; d Institute of Himalayan Risk Reduction, Lalitpur, Nepal; e Faculty of Science and Technology, Bournemouth University, Bournemouth, UK; f Centre for Midwifery & Women’s Health, Bournemouth University, Bournemouth, UK

**Keywords:** Natural hazards, disaster preparedness, institutional capacity, early warning systems, Nepal

## Abstract

Nepal faces many natural hazards, including floods, landslides, earthquakes, and extreme weather, which severely impact infrastructure, public health, and community resilience. In 2025, we conducted a formative stakeholder and community engagement exercise to inform research on strengthening local preparedness. This included consultative meetings with national agencies, local government, and community members in two hazard-affected municipalities. Field notes from the meetings were thematically analysed to identify priority themes. Findings indicated that national legal and policy frameworks exist, but implementation at municipal and ward levels remains inconsistent and hindered by limited institutional capacity. Local actions were largely focused on relief and reconstruction rather than preparedness and risk reduction. Community members and local officials described limited awareness of preventive strategies but strong willingness to engage in preparedness planning. Stakeholders identified priorities including improved coordination, legal literacy, locally tailored early-warning communication, and better data systems. These findings will inform co-development of a practical preparedness resource.

## PAPER CONTEXT

- **Main findings**: Local disaster preparedness in two Nepalese municipalities remained largely relief-oriented, despite national disaster risk management frameworks and community willingness to engage in preparedness.
- **Added knowledge**: Multi-level engagement identified practical implementation barriers, including limited legal literacy, weak coordination, low preparedness awareness, gaps in early-warning communication, and limited local loss-and-damage data systems.
- **Global health impact for policy and action**: Locally adaptable preparedness tools and inclusive community engagement can support risk reduction and strengthen disaster resilience in resource-limited settings.

## Background

Nepal is one of the most disaster-prone countries [1], with over 80% of people exposed to natural hazards annually [2,3]. The diverse topography, geography, and climate contribute to a wide range of hazard types: the mountains face extreme cold, avalanches, and glacial lake floods; the mid-hills are prone to landslides, lightning, floods, and forest fires; and the southern plains experience extreme temperature and recurrent monsoon flooding [4]. The 2015 earthquake resulted in nearly 9,000 deaths and over 22,000 injuries [5], while the 2024 floods and landslides claimed 215 lives, reminding us of recurring threats posed by natural hazards [6].

Natural hazard events disproportionately affect marginalised, rural, and remote communities by disrupting healthcare access and increasing the burden of physical injuries, non-communicable diseases, and mental health conditions such as post-traumatic stress disorder and depression [7]. Pregnant women, children, older adults, and persons with disabilities are particularly vulnerable. The impact of these events is intensified by climate change and environmental degradation [2,8]. Natural hazards often push families deeper into poverty through the loss of earnings, catastrophic health expenditure, or long-term care costs.

Following federalisation in 2015, the Disaster Risk Reduction and Management Act (2017) delegated disaster risk management (DRM) responsibilities across federal, provincial, and local governments [8,9]. While national departments for disaster and health emergency management have been established, a fully integrated and coordinated emergency medical response system remains limited. Local governments often lack resources, know-how, and coordination mechanisms in this field [9].

Community-based strategies are essential for building disaster resilience [2], as local capacity-building, public awareness, and integrating appropriate technology can help reduce disaster impacts [3]. However, community engagement in research is poorly utilised in many South Asian countries [10]. Initiatives such as the Nepal Risk Reduction Consortium in 2011 and the National Disaster Risk Reduction and Management Authority (NDRRMA) in 2019 have strengthened the policy environment. However, local implementation remains inconsistent, with significant gaps in operationalising national policies into practical, context-specific strategies [8].

The Sendai Framework for Disaster Risk Reduction (2015–2030) calls for localisation of DRM policies, yet Nepal has not succeeded in this [11]. To date, few studies have examined how national policy actors, municipal officials, and hazard-affected community members jointly understand the barriers to local preparedness. This short communication describes early, multi-level insights to inform the co-development of a locally usable preparedness resource.

## Approach

In March 2025, we conducted formative stakeholder and community engagement, not to evaluate existing programmes, but to identify priorities, barriers, and opportunities for developing locally relevant preparedness resources.

National stakeholders were purposively selected because of their roles in disaster risk reduction, hydrometeorological monitoring, health emergency response, and disaster preparedness research. Community engagement was conducted in two hazard-affected municipalities: one rural municipality in Sindhupalchok district (locality 1) and one in Kavrepalanchok district (locality 2). To establish a diverse group, officials and community contributors were invited through local contacts (Table 1).

**Table 1.** Summary of stakeholder and community engagement meetings.

Institution/Organisation	Representative/Contributors
Ministry of Home Affairs, Kathmandu	Senior decision-makers with responsibility for natural hazard management in National Disaster Risk Reduction and Management Authority (*n* = 1)
Ministry of Energy, Water Resources and Irrigation, Kathmandu	Senior decision-makers with responsibility for natural hazard management in Department of Hydrology & Meteorology (*n* = 3)
Ministry of Health and Population, Kathmandu	Senior decision-makers with responsibility for natural hazard management in Health Emergency Operations Centre (*n* = 4)
NGO (Institute of Himalayan Risk Reduction), Kathmandu	Senior members of the institute with extensive experience in natural hazard management (*n* = 2)
Government Officials Meeting locality 1	Mayor, Chief Administrative Officer, Health Coordinator, Sub Health Coordinator, Ward Chairpersons (Wards 1–3), Hospital Administrator, Medical Officers (In-Charge & Sub-In-Charge), Hospital Operator (*n* = 11)
Community Members Meeting locality 1	Women’s Groups, Youth (Child Club, Scouts), Red Cross, Older People, Female Community Health Volunteers, Persons with Disabilities, School Management Committee, Ward Officials, Police, Community Representatives (*n* = 18)
Government Officials Meeting locality 2	Mayor, Deputy Mayor, Chief Administrative Officer, Health Coordinator, Sub Health Coordinator, Public Health Inspector, Administrative Officer, Senior Division Engineer, Red Cross Representative, Senior Auxiliary Health Workers (*n* = 9)
Community Members Meeting locality 2	Ward Chair, Ward Secretary, Child Mobilizer, School Teachers, Child Club Members, Female Community Health Volunteers, Cooperative Members, Disaster Survivors (*n* = 24)

*Note:* Total participants across all stakeholder meetings and community engagement activities: n = 72.

Activities included consultative meetings, held in Nepali, with national agencies, local government, and community members. A semi-structured topic guide explored recent hazards, preparedness and response arrangements, awareness of relevant policies, early-warning communication, local capacity needs, and future research priorities. Field notes were analysed using a rapid thematic approach. Emerging themes were identified from field notes and reviewed by members of the research team to refine and agree the final themes.

Ethical approval exemption was granted by Kathmandu Medical College Institutional Review Committee, and verbal informed consent was obtained from all contributors.

## Findings

Stakeholder meetings and community engagement activities identified key themes relating to disaster preparedness, highlighting current strengths, challenges, and opportunities for strengthening local preparedness and response.

## National-level preparedness infrastructure

Centrally, the NDRRMA is actively functioning; it has developed legal instruments, plans, and strategies for a diverse range of hazards, and is conducting public awareness activities either independently or in partnership with stakeholders. Many activities are designed for, and disseminated to, local and provincial governments. Disaster Risk Reduction focal persons have been assigned across municipalities, provincial and federal departments/ministries, while Disaster Risk Reduction committees have been formalised down to the ward level.

## Local government

Local governments and agencies appear primarily focused on post-disaster relief and reconstruction. Local officials described challenges in translating legislation into practice because of limited technical capacity and funding, lack of trained focal persons, and inadequate operational guidance. Local policies were perceived to prioritise financial and material support after events, rather than proactive thinking.

## Community awareness and engagement

Some community contributors and local officials described events as beyond human control, reflecting limited awareness of risk reduction and preventive strategies. However, they were willing to engage in preparedness activities and showed awareness of human factors that affect disaster risk, including intentionally set wildfires, landslides linked to unregulated road construction and flooding associated with altered riverbeds.

## Coordination and implementation challenges

Local Disaster Management Committees have been established but lack capacity to operate effectively. Local government representatives responded positively to the idea of strengthening capacity. We also identified a strong need to educate local leaders (including elected representatives) and government staff about existing legislation and to promote public awareness.

Community contributors expressed frustration with delays in receiving post-disaster relief. Stakeholders recognised the importance of accurate data on disaster-related loss and damage, suggesting the need to incorporate simple data collection mechanisms in future preparedness activities.

## Discussion and implications

This paper informs a larger co-produced research programme and practical preparedness resource, rather than evaluating disaster risk management programmes. The findings highlight the need to strengthen local disaster preparedness and injury prevention, particularly by addressing the gap between national policy frameworks and local implementation. These priorities align with the Sendai Framework, which emphasises understanding disaster risk, strengthening disaster risk governance, and investing in resilience and preparedness [11].

*Bridging policy and practice gaps*: Although Nepal has comprehensive legal frameworks and strategies, implementation at local level remains inconsistent. This reflects wider implementation challenges in decentralised systems, requiring local capacity, resources, leadership, and practical delivery mechanisms. Simplified tools, practical training, institutionalization of practices, and sustained engagement with local leaders are needed.

*Institutionalising community engagement*: Regular and inclusive engagement with communities should be embedded within disaster risk management processes, since it can improve risk knowledge, local monitoring, communication, and response capacity [12].

*Prioritising risk reduction over response*: Local strategies largely focus on reactive post-disaster relief. Greater emphasis on climate change mitigation (e.g. land-use planning), identification and pro-active management of vulnerable populations and hazards (e.g. through early-warning systems, public awareness, and disaster planning) would support a shift towards evidence-based proactive risk reduction [13].

*Building integrated data systems for local action*: Many recognise the importance of reliable data for planning and advocacy. Simple, user-friendly, and locally maintained systems could track hazard type, location, deaths, injuries, displacement, infrastructure damage, response delays and relief needs. Such systems could strengthen local decision-making and support evidence-informed advocacy for resources.

*Leveraging intersectoral partnerships*: Enhanced coordination among central agencies, NGOs, and academic institutions can facilitate the flow of knowledge, resources, and innovations to local levels. Partnerships that integrate public health, engineering, and social science perspectives will support resilient systems [14].

### Implications for future research

Future research should focus on developing scalable, context-specific resources that support Nepal’s local and provincial governments in enhancing disaster preparedness and response. These resources could include preparedness checklists, action-planning templates, tailored early-warning communication materials, simple tools for recording disaster-related injuries, deaths and losses, and training packages for elected representatives, local officials, and community groups. Given their autonomy and closer proximity to communities, local governments are well-positioned to implement timely and targeted interventions. However, they require evidence-informed tools, capacity-building strategies, and systems-thinking approaches to plan effectively for diverse hazards.

Studies should prioritise the co-creation of preparedness frameworks that are grounded in community realities and aligned with national disaster management goals. Specific research questions emerging from this engagement include: how can national disaster risk management policies be translated into practical municipal and ward-level actions; how can vulnerable groups be meaningfully included in preparedness planning; and how can local data, early-warning information and community knowledge be integrated into routine decision-making?

Community engagement should be central to future research. Human-Centred Design methods may be particularly useful since they support iterative co-design with end-users, helping to ensure that preparedness tools are practical, acceptable, locally relevant, and feasible within existing governance systems [15].

Future research should also explore how these tools can be institutionalised through municipal planning, budgeting, ward-level DRM committees, designated focal persons, and routine training. Barriers such as limited resources, staff turnover, unclear mandates and weak coordination across health, disaster management, and local government systems should also be examined.

## Limitations

Our activities were conducted in only two localities at one time point, so findings are not representative of the country. As sessions were designed as formative engagement rather than formal qualitative interviews, discussions were not audio-recorded nor fully transcribed, limiting detailed comparison between localities and gender- and age-disaggregated analysis. The findings, therefore, provide preliminary insights to inform future co-developed research and intervention design.

## Conclusions

We have highlighted a persistent gap between Nepal’s national disaster risk management frameworks and their practical implementation at local levels. By bringing together national, local government, and community perspectives, we have identified practical priorities for strengthening preparedness: locally adaptable tools, clearer coordination, legal literacy, inclusive community engagement, tailored early-warning communication, and routine loss-and-damage data. The findings provide a useful foundation for co-developing and testing the feasibility of a municipal preparedness resource.

## Data Availability

The engagement materials generated during this work consist of field notes and meeting summaries. These are not publicly available because contributors did not provide consent for public data sharing and the notes may contain contextual information that could identify specific communities, institutions, or local stakeholders.

## References

[1] Jimee GK, Meguro K, Dixit AM. Nepal, a multi-hazard risk country: spatio-temporal analysis. J Nepal Geol Soc. 2019;58:145–5. doi: 10.3126/jngs.v58i0.24599

[2] Joshi BR, Pandey P, Tiwari K, et al. Disaster management policies in Nepal: current status and way forward. Int J Adv Trends Eng Technol. 2021;6:42–52.

[3] Ministry of Health and Population. Health emergency operation center network of Nepal: The voyage and the vista [Internet]. Kathmandu: Government of Nepal; 2021 [cited 2025 Oct 26]. Available from: https://heoc.mohp.gov.np/guidelines-publications/health-emergency-operation-center-network-of-nepal-the-voyage-and-the-vista/detail

[4] Kato N, Shaw R. Evolution of disaster risk governance and its implication to resilience building in Nepal. Dis Prev Res. 2024;3:7. doi: 10.20517/dpr.2024.05

[5] National Planning Commission. Nepal earthquake 2015: post disaster needs assessment vol. A: key findings. Kathmandu: Government of Nepal, National Planning Commission; 2015 [cited 2026 Jun 14]. Available from: https://www.worldbank.org/content/dam/Worldbank/document/SAR/nepal/PDNA%20Volume%20A%20Final.pdf

[6] United Nations. Nepal: hundreds killed as ‘unprecedented’ flash floods strike capital Kathmandu [Internet]. UN News. 2024 [cited 2025 Oct 25]. Available from: https://news.un.org/en/story/2024/10/1155246

[7] Keya TA, Leela A, Habib N, et al. Mental health disorders due to disaster exposure: a systematic review and meta-analysis. Cureus. 2023;15:e37031. doi: 10.7759/cureus.37031 37143625 PMC10153020

[8] Khanal BN. Nepal: a brief country profile on disaster risk reduction and management [Internet]. Kobe: Asian Disaster Reduction Center; 2020 [cited 2025 Oct 26]. Available from: https://www.adrc.asia/countryreport/NPL/2019/Nepal_CR2019B.pdf

[9] Bhandari D, Neupane S, Hayes P, et al. Disaster risk reduction and management in Nepal: delineation of roles and responsibilities [Internet]. Oxford: Oxford Policy Management; 2020 [cited 2025 Oct 26]. Available from: https://www.opml.co.uk/files/Publications/a1594-strengthening-the-disaster-risk-response-in-nepal/delineation-of-responsibility-for-disaster-management-full-report-english.pdf

[10] Simkhada B, van Teijlingen E, Naheed A, et al. Importance of involving patients and public in health research in Bangladesh and Nepal. Int J Technol Assess Health Care. 2021;37:e10. doi: 10.1017/S0266462320000811 33150862

[11] Ministry of Home Affairs. Review of the implementation of Sendai framework for disaster risk reduction 2015–2030: Nepal national voluntary report [Internet]. Kathmandu: Ministry of Home Affairs, Government of Nepal; 2022 [cited 2025 Oct 15]. Available from: https://sendaiframework-mtr.undrr.org/media/87427/download?startDownload=20250506

[12] Sufri S, Dwirahmadi F, Phung D, et al. A systematic review of community engagement in disaster early warning systems. Prog Disaster Sci. 2020;5:100058.

[13] Tome J, Richmond HL, Rahman M, et al. Climate change and health vulnerability in Nepal: a systematic review of the literature since 2010. Glob Public Health. 2022;17:1406–1419. doi: 10.1080/17441692.2021.1924824 34061709

[14] Gooding K, Bertone MP, Loffreda G, et al. How can we strengthen partnership and coordination for health system emergency preparedness and response? Findings from a synthesis of experience across countries facing shocks. BMC Health Serv Res. 2022;22:1441. doi: 10.1186/s12913-022-08859-6 36447261 PMC9706990

[15] Kang BA, Poddar M, Luitel A, et al. Narrative review of human-centered design in public health interventions in low-and middle-income countries: recommendations for practice, research, and reporting. Glob Health Sci Pract. 2025;13:e2400164. doi: 10.9745/GHSP-D-24-00164 40813242 PMC12352946

